# Prognostic interaction between bone marrow morphology and *SF3B1* and *ASXL1* mutations in myelodysplastic syndromes with ring sideroblasts

**DOI:** 10.1038/s41408-018-0051-1

**Published:** 2018-02-12

**Authors:** Abhishek A. Mangaonkar, Terra L. Lasho, Christy M. Finke, Naseema Gangat, Aref Al-Kali, Michelle A. Elliott, Kebede H. Begna, Hassan Alkhateeb, Alexandra P. Wolanskyj-Spinner, Curtis A. Hanson, Rhett P. Ketterling, William J. Hogan, Animesh Pardanani, Mark R. Litzow, Ayalew Tefferi, Mrinal M. Patnaik

**Affiliations:** 1 Department of Hematology, Mayo Clinic, Rochester, USA; 2 Department of Pathology, Mayo Clinic, Rochester, USA

The 2016 iteration of the World Health Organization (WHO) classification of myeloid neoplasms includes myelodysplastic syndrome with ring sideroblasts (MDS-RS) as a unique sub-type of MDS. Presence of dysplasia in single or multiple cell lineages with at least 15% ring sideroblasts (RS), or as few as 5% RS in the presence of *SF3B1* mutations, further subcategorizes “MDS-RS” into: MDS-RS with single lineage dysplasia (SLD) and MDS-RS with multi-lineage dysplasia (MLD)^1^. In the prior 2008 WHO classification, MDS-RS-SLD was termed as refractory anemia with ringed sideroblasts and MDS-RS-MLD was included under refractory cytopenia with multi-lineage dysplasia, a category that also incorporated MDS patients with MLD without RS^2^. These changes in classification were prompted on the basis of morphological distinctions and their impact on prognosis. In addition, the strong phenotypic correlation of *SF3B1* mutations with bone marrow (BM) RS^3^ and the prognostic irrelevance of BM RS percentage^4^, resulted in the inclusion of these mutations in the classification schema. The advent of next generation sequencing has established the molecular landscape in MDS, with frequent gene mutations including *SF3B1* (20–30%), *TET2* (~20%), *ASXL1* (~14–15%), and *RUNX1* (~8–9%)^5–7^. We pursued this study to assess the impact of degree of bone marrow dysplasia, in the context of gene mutations in patients with WHO defined MDS-RS.

Successive cases of MDS-RS, meeting the 2016 WHO criteria were identified from our institutional database from years 1994 to 2015. All patients had BM biopsies & cytogenetics at diagnosis. BM slides including Prussian blue stains for RS were re-reviewed to ensure compliance with the WHO criteria. Targeted exome sequencing for the following genes; *TET2, ASXL1, DNMT3A, IDH1, IDH2, TP53, SRSF2, SF3B1, SH2B3, NPM1, FLT3, U2AF1, ZRSR2, JAK2, CSF3R, MPL, MFSD11, CEBPA, SETBP1, ZRSR2, RUNX1, IKZF1, CALR, KRAS, NRAS, CBL, PTPN11, STAG2, BCOR*, and *GATA2*, was performed on diagnostic BM specimens from 64 patients by previously described methods^8^. Detailed clinical, laboratory and treatment data was collected. Mann–Whitney and Fisher exact test was used to assess differences among numerical and categorical variables between the SLD and MLD groups respectively. Multivariate analysis was performed using the Cox Proportional Hazards model. Statistics were performed via JMP Pro software (version 10).

Seventy six patients with MDS-RS met the study criteria, median age 73 years (range: 44–88), 50 (66%) males. Of these 57 (75%) were categorized as MDS-RS-SLD and 19 (25%) as MDS-RS-MLD. Six (8%) patients had an abnormal karyotype, which were further stratified into R-IPSS one (1.32%) intermediate, four (5.26%) poor, and one (1.32%) very poor cytogenetic risk groups. Overall R-IPSS stratification included 2 (2%) very low, 40 (53%) low, 22 (29%) intermediate, 9 (12%) high, and 3 (4%) very high-risk categories respectively. Targeted exome sequencing was available in 64 cases and demonstrated the following mutational frequencies; *SF3B1* 77%, *ASXL1* 16%, *DNMT3A* 13%, *TET2* 6%, *TP53 5%*, *IDH1* 3, and 2% each for *SRSF2*, *PTPN11*, *ZRSR2*, *CSF3R*, and *U2AF1* (see Table 1).

**Table 1:** Table displaying distribution of variables between MDS-RS, MDS-RS-SLD, and MDS-RS-MLD

*Variable; median value (range or %)*	MDS-RS (*n* = 76)	SLD (*n* = 57)	MLD (*n* = 19)	*P-value*
Age (years)	73 (44–88)	73 (47–88)	72 (44–81)	0.22
No. of males	50 (66)	36 (63)	14 (74)	0.4
Hb; gm/dl	9.3 (5.8–14.4)	9.3 (5.8–13.4)	10 (7.8–14.4)	0.17
WBC count x 10^9^ per liter	5.1 (1.2–17.6)	5.7 (2.2–17.6)	4.4 (1.2–9.4)	0.02 *
ANC x 10^9^ per liter	3.06 (0.3–12.5)	3.2 (0.6–12.5)	2.13 (0.3–6.3)	0.02 *
Platelet count x 10^9^ per liter	237 (7–819)	255 (7–819)	202 (27–438)	0.05 *
BM ringed sideroblasts	35 (10–80)	50 (10–80)	20 (15–70)	0.004 *
BM blasts	1(0–4)	1(0–4)	1(0–3)	0.6
*Cytogenetics*				
Abnormal karyotype (R-IPSS intermediate, poor and very poor cytogenetic risk groups)	6 (8)	4 (8)	2 (11)	0.5
*IPSS cytogenetic risk groups*				
Good	69 (91)	52 (91)	17 (11)	0.5
Intermediate	2 (3)	2 (4)	0(0)	
Poor	5 (6)	3 (5)	2 (11)	
*R-IPSS cytogenetic risk groups*				
Very good	2 (3)	1 (2)	1 (5)	0.6
Good	67 (88)	51 (89)	16 (84)	
Intermediate	2 (3)	2 (4)	0(0)	
Poor	4 (5)	2 (4)	2 (11)	
Very poor	1 (1)	1 (2)	0(0)	
*Genomic abnormalities*				
*SF3B1*	49 (77)	41 (82)	8 (57)	0.06
*ASXL1*	10 (16)	6 (12)	4 (29)	0.15
*DNMT3A*	8 (13)	8 (16)	0(0)	0.04 *
*TET2*	4 (6)	4 (8)	0(0)	0.2
*TP53*	3 (5)	3 (6)	0(0)	0.2
*IDH1*	2 (3)	2 (4)	0(0)	0.3
*SRSF2*	1 (2)	1 (2)	0(0)	–
Others (*PTPN11*, *ZRSR2, CSF3R*, *U2AF1*)	4 (6)	4 (8)	0(0)	–
*Treatment*				
HMA treatment	5 (7)	2 (4)	3 (16)	0.2
Immunomodulatory treatment (Lenalidomide)	1 (1)	1 (2)	0(0)	0.83
Allogeneic HSCT	2 (3)	1 (2)	1 (5)	0.16
*Outcomes*				
Leukemic transformation	2 (3)	1 (2)	1 (5)	0.34
Overall survival; median months (range)	46 (0–190)	47 (0–190)	44 (6–123)	0.2

* Signify statistically significant values

In comparison to MDS-RS-MLD, patients with MDS-RS-SLD had a higher frequency of *SF3B1* (82 vs 57%, *p* = 0.06) and *DNMT3A* (16 vs 0%, *p* = 0.04) mutations, however the frequency of *ASXL1* (12 vs 29%, *p* = 0.15) mutations was lower. At a median follow-up of 33 months, 68 (89%) deaths and 2 (3%) leukemic transformations were documented. Median survival (OS) of the entire cohort was 46 months (Range: 0–190 months). Survival for the MDS-RS-SLD group was 47 months, while that for MDS-RS-MLD was 44 months [Hazard ratio (HR) 1.4, *p* = 0.2]. On a univariate survival analysis that included age, sex, hemoglobin, white blood cell count with individual differential counts, platelet count, BM morphology (SLD versus MLD), peripheral blood blasts, cytogenetics and the aforementioned gene mutations, lack of *SF3B1* mutations (HR 4.35, 95% CI 2.2–8.3, *p* < 0.0001, Fig. 1a) and presence of *ASXL1* mutation (HR 3.13, 95% CI 1.4-6.3, *p* = 0.006, Fig. 1b) adversely impacted OS. Interestingly, multilineage dysplasia did not show a statistically significant correlation with outcomes (HR 1.4, 95% CI 0.8–2.5, *p* = 0.2) (Fig. 1c). In a multivariate analysis that included *ASXL1* and *SF3B1* mutations as variables, both the presence of *ASXL1* mutations [HR of 2.3 (95% CI 1.0–4.7), *p* = 0.05] and the absence of *SF3B1* mutations [HR 3.7 (95% CI 1.8–7.2), *p* = 0.0006] retained an independent and negative prognostic impact. In addition, both these mutations retained their prognostic impact when analyzed in the context of R-IPSS risk categories. Further, we assessed the combined effect of presence (mutated or mt) or absence (wild-type or wt) of *ASXL1* and *SF3B1* mutations on outcomes. Median OS was longest in the *ASXL1*wt/*SF3B1*mt (*n* = 42, 69 months) sub-group of patients in comparison to others (*p* < 0.0001), suggesting that the presence of both these mutations need to be assessed at diagnosis for an accurate prognostication (Fig. 1d). Patient numbers were limited for individual comparison with other sub-groups.

**Fig. 1:**
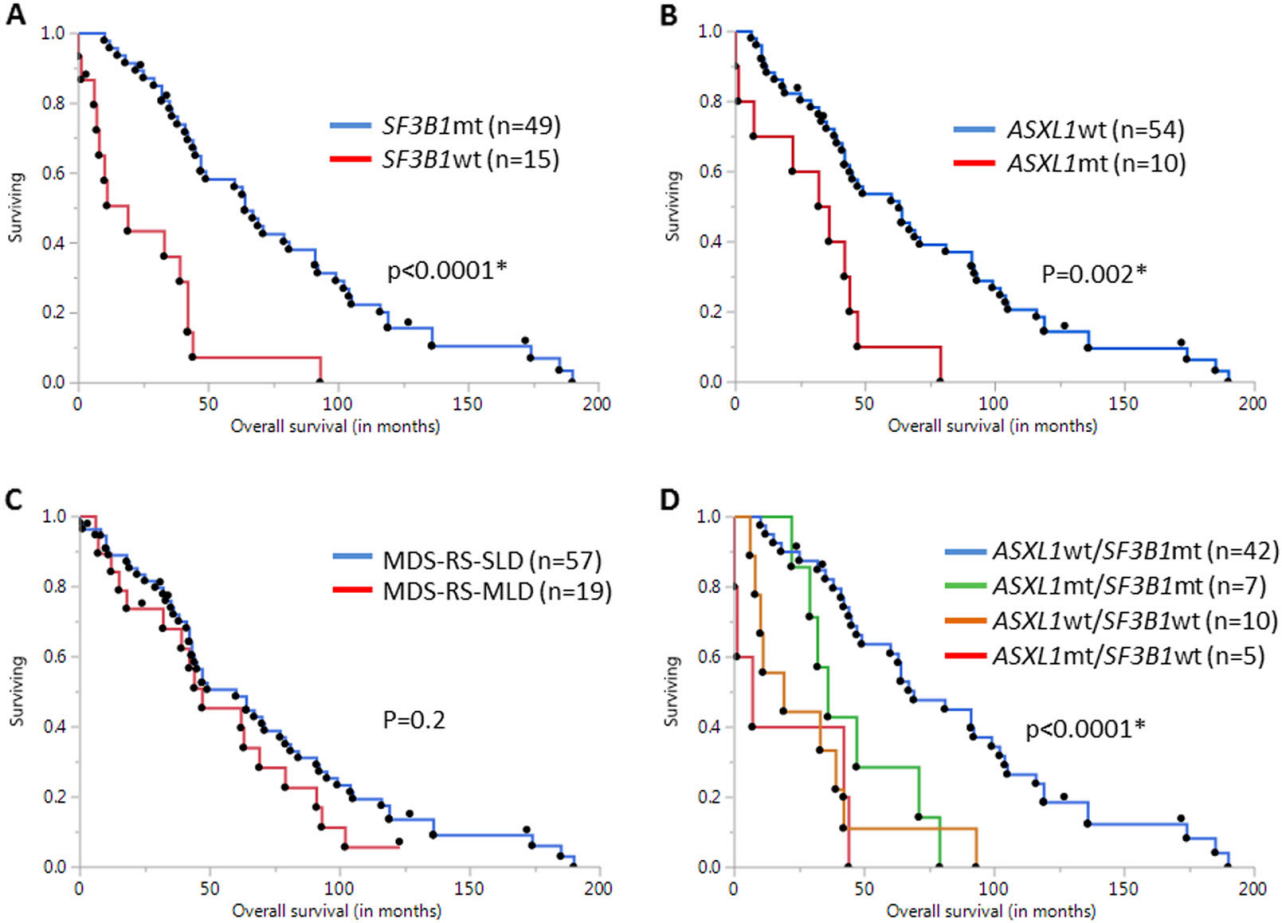
Kaplan-Meir survival analysis for MDS-RS patients based on somatic mutational analysis. **a** shows Kaplan-Meir survival curves for *SF3B1* mutated (mt) and *wild-type* (wt) patients. Log-rank test was used to assess difference between groups. Median OS is significantly higher in the *SF3B1* mt patients (64 versus 19 months, *p* < 0.0001*). **b** shows a similar analysis, depending on *ASXL1* mutational status. Median OS in *ASXL1* wt patients is much higher than *ASXL1* mt patients (64 versus 39 months, *p* = 0.002*). **c** shows Kaplan–Meir survival curves among the two sub-groups based on bone marrow morphology (MDS-RS-SLD and MDS-RS-MLD). No significant survival difference was found between the two groups (*p* = 0.2). **d** shows Kaplan-Meir survival curves for four sub-groups, based on presence or absence of both *SF3B1* and *ASXL1* mutations. Median OS was highest in *ASXL1* wt/*SF3B1* mt (*n* = 42, 69 months) sub-group (*p* < 0.0001*), followed by *ASXL1* mt/*SF3B1* mt (*n* = 7, 39 months), *ASXL1* wt/*SF3B1* wt (*n* = 10, 19 months) and *ASXL1* mt/*SF3B1* wt (*n* = 5, 7 months) sub-groups, respectively

Incorporation of genomic alterations into existing WHO morphological classifications is critical for better classifying these disorders and to refine existing prognostic strategies^9^. Our study validates the prognostic impact of gene mutations in MDS-RS and also assesses their relevance in the context of existing morphological distinctions.

Mutations in *SF3B1*, a gene regulating pre-mRNA splicing, are known to have favorable outcomes in MDS-RS^3,10^ and our study has confirmed this observation. In contrast, *ASXL1* mutations impact chromatin regulation by impairing activity of polycomb repressive complex 2 (PRC2), and are associated with adverse outcomes in myeloid neoplasms such as MDS, chronic myelomonocytic leukemia and myelofibrosis^8,11,12^. To our knowledge, ours is the first study to assess the prognostic impact of *ASXL1* mutations in an independent large cohort of WHO-defined MDS-RS patients and has demonstrated an adverse impact on survival. In particular, the subgroup of MDS-RS patients with mutated *SF3B1* and wild-type *ASXL1* has the best prognosis. In conclusion, molecular abnormalities involving relevant genes take precedence in terms of prognostication within morphologically defined subsets of MDS and in fact, may be more relevant than existing morphological prognosticators such as degree of bone marrow dysplasia. Collaborative prospective efforts with larger number of patients are needed to validate these findings.

## References

[1] Arber DA (2016). The2016 revision to the World Health Organization classification of myeloid neoplasms and acute leukemia. Blood.

[2] Vardiman JW (2009). The2008 revision of the World Health Organization (WHO) classification of myeloid neoplasms and acute leukemia: rationale and important changes. Blood.

[3] Patnaik MM (2012). SF3B1 mutations are prevalent in myelodysplastic syndromes with ring sideroblasts but do not hold independent prognostic value. Blood.

[4] Patnaik MM (2012). Prognostic irrelevance of ring sideroblast percentage in World Health Organization-defined myelodysplastic syndromes without excess blasts. Blood.

[5] Bejar R (2011). Clinical effect of point mutations in myelodysplastic syndromes. N. Engl. J. Med..

[6] Papaemmanuil E (2011). Somatic SF3B1 mutation in myelodysplasia with ring sideroblasts. N. Engl. J. Med..

[7] Patnaik MM, Tefferi A (2017). Refractory anemia with ring sideroblasts (RARS) and RARS with thrombocytosis (RARS-T): 2017 update on diagnosis, risk-stratification, and management. Am. J. Hematol..

[8] Patnaik MM (2016). Prognostic interaction between ASXL1 and TET2 mutations in chronic myelomonocytic leukemia. Blood. Cancer J..

[9] Tefferi A (2017). Targeted next-generation sequencing in myelodysplastic syndromes and prognostic interaction between mutations and IPSS-R. Am. J. Hematol..

[10] Malcovati L (2015). SF3B1 mutation identifies a distinct subset of myelodysplastic syndrome with ring sideroblasts. Blood.

[11] Bejar R (2012). Validation of a prognostic model and the impact of mutations in patients with lower-risk myelodysplastic syndromes. J. Clin. Oncol..

[12] Thol F (2011). Prognostic significance of ASXL1 mutations in patients with myelodysplastic syndromes. J. Clin. Oncol..

